# Modeling the CO_2_-effects of forest management and wood usage on a regional basis

**DOI:** 10.1186/s13021-015-0024-7

**Published:** 2015-06-12

**Authors:** Marcus Knauf, Michael Köhl, Volker Mues, Konstantin Olschofsky, Arno Frühwald

**Affiliations:** 1 Knauf Consulting, Dorotheenstrasse 7, Bielefeld, D-33615 Germany; 2 University of Hamburg (Center for Wood Sciences – World Forestry), Leuschnerstrasse 91, Hamburg,, D-21031 Germany; 3 University of Hamburg (Center for Wood Sciences – Mechanical Technology), Leuschnerstrasse 91, Hamburg,, D-21031 Germany

**Keywords:** Climate protection, Modeling, Scenario analysis, Carbon stock, Emission reduction through substitution, Carbon management in forestry and wood products

## Abstract

**Background:**

At the 15^th^ Conference of Parties of the UN Framework Convention on Climate Change, Copenhagen, 2009, harvested wood products were identified as an additional carbon pool. This modification eliminates inconsistencies in greenhouse gas reporting by recognizing the role of the forest and timber sector in the global carbon cycle. Any additional CO_2_-effects related to wood usage are not considered by this modification. This results in a downward bias when the contribution of the forest and timber sector to climate change mitigation is assessed. The following article analyses the overall contribution to climate protection made by the forest management and wood utilization through CO_2_-emissions reduction using an example from the German state of North Rhine-Westphalia. Based on long term study periods (2011 to 2050 and 2100, respectively). Various alternative scenarios for forest management and wood usage are presented.

**Results:**

In the mid- to long-term (2050 and 2100, respectively) the net climate protection function of scenarios with varying levels of wood usage is higher than in scenarios without any wood usage. This is not observed for all scenarios on short and mid term evaluations.

The advantages of wood usage are evident although the simulations resulted in high values for forest storage in the C pools. Even the carbon sink effect due to temporal accumulation of deadwood during the period from 2011 to 2100 is outbalanced by the potential of wood usage effects.

**Conclusions:**

A full assessment of the CO_2_-effects of the forest management requires an assessment of the forest supplemented with an assessment of the effects of wood usage. CO_2_-emission reductions through both fuel and material substitution as well as CO_2_ sink in wood products need to be considered.

An integrated assessment of the climate protection function based on the analysis of the study’s scenarios provides decision parameters for a strategic approach to climate protection with regard to forest management and wood use at regional and national levels.

The short-term evaluation of subsystems can be misleading, rendering long-term evaluations (until 2100, or even longer) more effective. This is also consistent with the inherently long-term perspective of forest management decisions and measures.

## Background

Forests play a significant role in the global carbon cycle. They sequester CO_2_ from the atmosphere through the process of photosynthesis and store the carbon over the long term. In the philosophy of UN-FCCC the act of increasing a forest’s carbon stock by increasing levels of standing biomass volumes is recognized as an emissions reduction measure. Stored carbon is released through biological decomposition in the forest back into the atmosphere mainly in the form of CO_2_; only a smaller fraction of the carbon is ultimately stored in the soil.

National reporting on greenhouse-gas emissions in the "Land Use, Land Use Change and Forestry (LULUCF)“sector includes an assessment of carbon sequestration by forests, cf. [1, 2]. The GPG LULUCF assumed that “all carbon removed in wood or other biomass from forests is oxidized in the year of removal” and released into the atmosphere [3]. This assumption did not consider the fact that when timber is harvested, no immediate CO_2_ emissions occur [4, 5], instead the carbon remains stored in the harvested wood products [6–9]. The Conference of the Parties to the Kyoto Protocol in Copenhagen, 2009, recognized the importance of including harvested wood products as carbon sinks in national greenhouse-gas reporting [10]. The conferences in Durban 2011 and Doha 2012 decided that carbon stored in harvested wood products would be integrated into the reporting by means of a forest management reference level (FMRL) [11].

In addition to the carbon storage function of harvested wood products, wood usage contributes to a reduction in CO_2_ emissions through so-called substitution effects:

1. Fuel substitution: wood replaces fossil fuels such as oil, gas or coal. The use of wood for energy is considered CO_2_-neutral, simplified by omitting the effects earlier in the value chain such as forest management, transport and manufacturing [12–14].
2. Material substitution: A relatively significant emission-savings effect results from using wood products in place of products made from other materials, such as concrete, plastic or steel. These non-wood products typically require more energy for their production and disposal and therefore generate higher CO_2_-emission levels than wood products [15–18].

In contrast to the temporary carbon storage function of forests and wood products, both the fuel and the material substitution effect of wood usage have a permanent impact on the reduction of atmospheric CO_2_. Extensive research exists on the fuel and material substitution effect of wood usage, e.g., [12, 13, 15, 19–22].

Until recently, the climate protection function of forests and wood usage was typically recorded separately for the forest management and wood-products industries. The analysis of subsystems can result in opposing recommendations for “optimal” forest management measures. While, for example, Nabuurs et al. [23] support timber harvesting, Luyssaert et al. [24] recommend longer rotation periods and no wood usage.

This study analyzes different scenarios to assess which forest management strategy has the greatest impact on emissions reduction. Applying an integrated approach, it takes into account the interactions between sequestration through forest growth, changes in carbon stocks in the forest and the harvested wood products pool through timber harvest and the manufacturing and use of wood products as well as the substitution effect of wood usage.

## Results

The results of the scenario analysis are presented for two components, first for the forest itself, and then for wood usage; the carbon sink function of the forest and the CO_2−_effects of wood usage are shown separately. For the sake of clarity, and in contrast to typical IPCC reporting, atmospheric CO_2_ sequestration and CO_2_ emissions reduction are designated as positive values.

### Scenario Analysis Forest Management

The **basic scenarios**, Volume strategy, value strategy and carbon storage strategy each impact timber growth differently, leading to differences in carbon stock and timber harvest levels. Table 1 shows the average annual change in forest carbon stocks (C-pools of belowground biomass, aboveground biomass and deadwood) determined using the stock change method for the study period from 2011 to 2100. It also shows the average annual timber harvest per hectare of forest area for the same time period. Over the 90 year period, forest carbon stocks increased most in the carbon storage strategy scenario with an average gain of 1.65 tC/ha and year. The value strategy scenario shows the lowest increase in forest carbon stocks at an average of 0.14 tC/ha and year. In the volume strategy scenario, designed for the highest level of timber harvest, the forest carbon stocks increased on average by 0.75 tC/ha and year.

**Table 1:** Annual change in forest carbon storage and annual timber harvest for the basic scenarios for North Rhine-Westphalia (2011–2100)

Per hectare	Volume strategy [tC/ha, a]	Value strategy [tC/ha, a]	Carbon storage strategy [tC/ha, a]
Average annual change in the forest carbon storage (2011–2100)	0.75	0.14	1.65
Average annual timber harvest (2011–2100)	3.22	3.00	1.64
Total	3.96	3.14	3.29

The volume and value strategy scenarios show harvest levels of 3.22 tC/ha or 3.00 tC/ha per year, respectively, while harvest levels in the carbon storage optimization scenario are significantly lower at 1.65 tC/ha per year. Considering the sum of timber harvest and forest carbon storage levels, the volume strategy scenario with 3.96 tC/ha per year has the greatest sequestration impact; the value strategy and carbon storage strategy scenarios differed only slightly (3.14 tC/ha and 3.29 tC/ha per year, respectively).

**Fig. 1:**
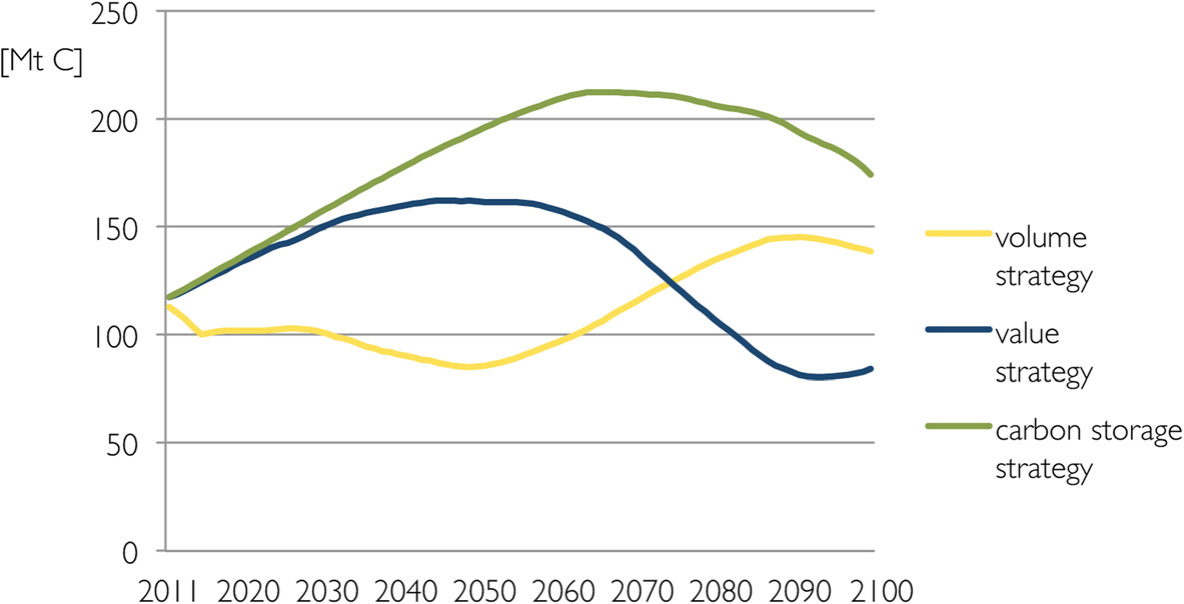
Changes in aboveground carbon stocks for the basic scenarios (2011–2100)

Figure 1 shows the development of the aboveground biomass for the three basic scenarios to the year 2100. The volume strategy scenario shows an early drop in carbon storage levels resulting from timber harvesting, followed by relatively constant level of aboveground biomass in the first half of the study period and an increase in timber stock levels during the second half of the study period. The opposite trend can be seen in the value strategy scenario where forest stands are harvested later in the study period. The carbon storage strategy scenario shows a continuous stock increase, which decreases later in the study period as a result of relatively low levels of timber harvesting.

**Fig. 2:**
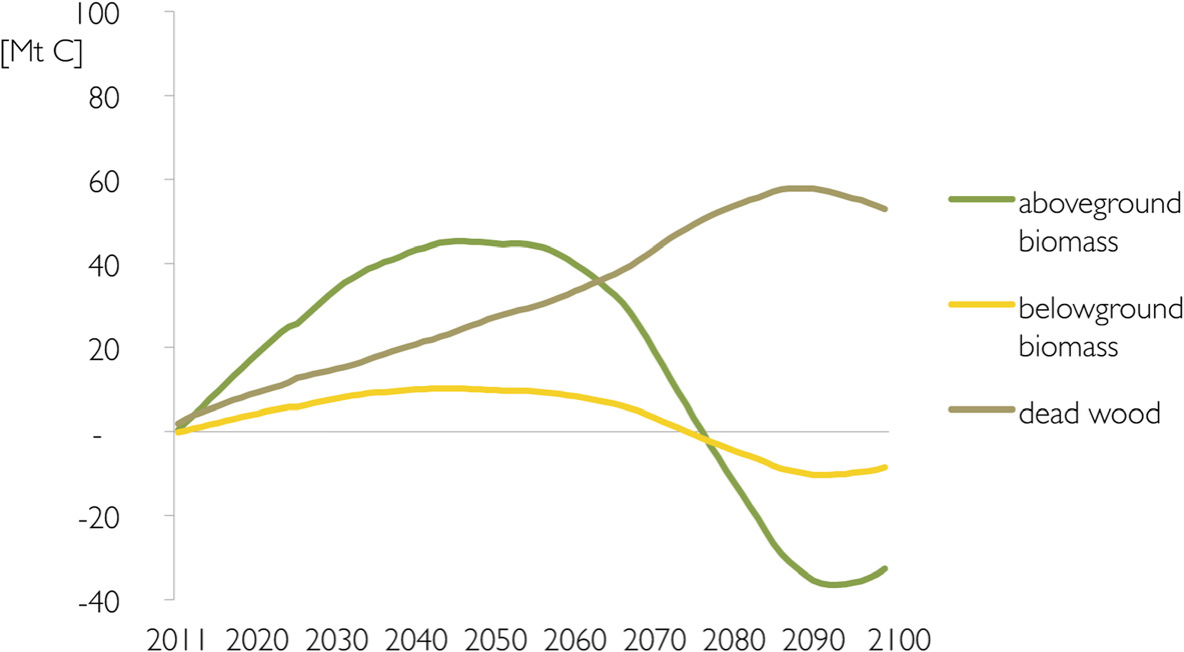
Changes in carbon stock levels of aboveground biomass, belowground biomass, and deadwood in the forest from 2011 to 2100 for the basic scenario value strategy

**Fig. 3:**
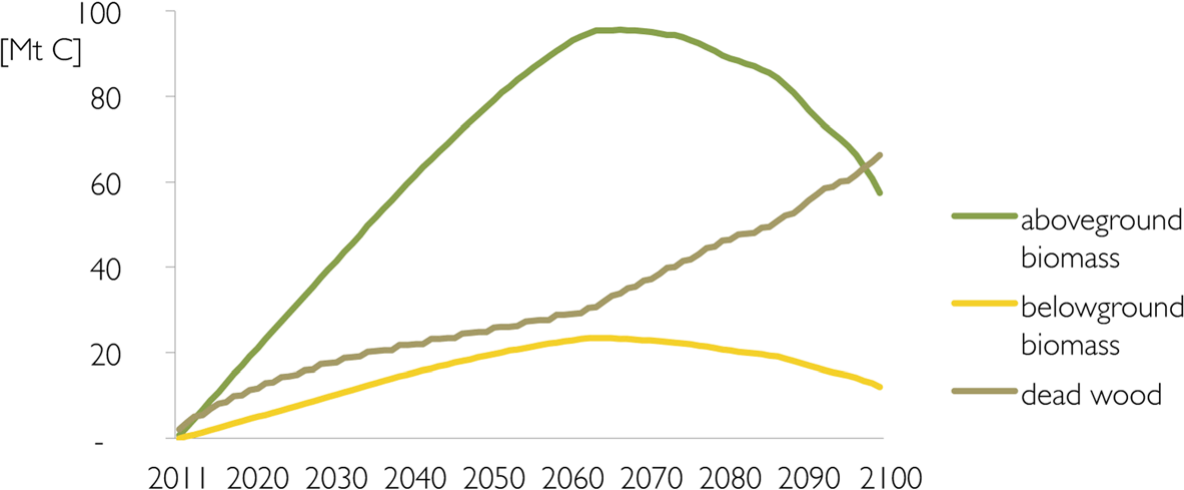
Changes in carbon stock levels of aboveground biomass, belowground biomass, and deadwood in the forest from 2011 to 2100 for the basic scenario carbon storage strategy

Figures 2 and 3 show carbon pool changes for the two basic scenarios of value strategy and carbon storage strategy comprised of the C-pools formed by aboveground biomass, belowground biomass and deadwood. Carbon storage in deadwood increases throughout the 90 year study period whereas C-pools in aboveground and belowground biomass decrease after reaching maximum levels in the middle of the period.

In the value strategy scenario, 20 % of harvested timber remains in the forest as deadwood. The deadwood C-pool continues to increase up to the year 2088, the production of deadwood overbalances the modeled decomposition of the deadwood. After the accumulation phase, during which the carbon stocks of the aboveground and belowground biomass increase, a phase of increased harvest activities follows that results in a decline in both C-pools. At the end of the study period, the carbon stock levels of the aboveground and belowground biomass stand well below the initial levels. Given the accumulation of deadwood, the total of all three C-pools in 2100 is greater than the initial level in 2011. Overall approximately 265 MtC were removed with harvested timber (3.00 tC/ha per year, see Table 1).

In the carbon storage strategy scenario the C-pools of aboveground and belowground biomass culminate in the year 2066 to the total maximal of 119 MtC, decrease thereafter to a level of 69 MtC in 2100 due to higher harvesting levels. The deadwood C-pool increases to 66 MtC in the year 2100, which is comparable to the levels for the C-pools of aboveground and belowground biomass. A total of approximately 135 MtC (1.64 tC/ha per year; see Table 1) is harvested as timber.

**Fig. 4:**
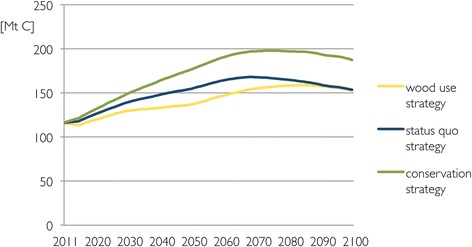
Carbon stock development of aboveground biomass for the combined scenarios (2011–2100)

The **combined scenarios** show lower variation over time and differences in carbon levels in comparison to the basic scenarios (Table 2, Fig. 4). The levels of carbon stocks from both, timber stocks and timber harvest levels (Table 2), reflect the averaging over the basic scenarios. The levels also take into account the various proportions of unused forest area of 5 or 10 %, respectively. Differences do exist though when comparing carbon stocks and timber harvest levels. While the level of carbon storage in the forest in the combined scenario “conservation strategy” is higher than in the combined scenarios “wood use strategy” and “status quo strategy”, there is a lower amount of timber available for harvest in the conservation strategy scenario.

**Table 2:** Annual change in forest carbon stocks and annual timber harvest levels for the combined scenarios for NRW (2011–2100)

Per hectare	Wood use strategy [tC/ha, a]	Status quo strategy [tC/ha, a]	Conservation strategy [tC/ha, a]
Average annual change in forest carbon stocks (2011–2100)	0.92	0.98	1.36
Average annual timber harvest (2011–2100)	2.69	2.49	2.06
Total	3.60	3.46	3.42

### Scenario analysis wood usage

The C-pools for harvested wood products, material substitution and fuel substitution were modeled for the harvested timber. The timber volumes are derived from the forest management scenarios in Table 8 and Table 9. The effects of wood usage are shown for the study period 2011–2050 in Table 3 and for the period 2011 to 2100 in Table 4, together with the results for the corresponding forest carbon stock levels. The C- or CO_2_-effects of wood usage (increase in the harvested wood products pool, emissions reduction through fuel and material substitution) are particularly high in periods with higher timber harvest levels (at the expense of the forest carbon pool), because the CO_2_ benefits of material substitution, extended carbon storage life in finished wood products and energy substitution of energy used by-products goes hand in hand with the simultaneous usage of the wood. This means that the volume strategy scenario which records a greater amount of wood usage early in the study period also shows greater C- or CO_2_-effects from wood usage. The carbon storage strategy scenario which records lower timber harvest levels earlier in the study period also shows lower C- or CO_2_-effects from wood usage; in this case wood usage was delayed to a later date when the stand reached higher maturity which resulted in a delay in C- or CO_2_-effects.

**Table 3:** Average annual C-effect of forest management and wood usage for the basic scenarios (2011–2050)

	Forest carbon stock [tC/ha, a]	HWP carbon stock [tC/ha, a]	Fuel substitution [tC/ha, a]	Material substitution [tC/ha, a]	Total [tC/ha, a]
Volume strategy	−0.3	0.9	1.5	2.5	4.7
Value strategy	2.2	0.3	0.9	1.3	4.8
Carbon storage strategy	3.4	0.0	0.4	0.7	4.4

**Table 4:** Average annual C-effect of forest management and wood usage for the basic scenarios (2011–2100)

	Forest carbon stock [tC/ha, a]	HWP carbon stock [tC/ha, a]	Fuel substitution [tC/ha, a]	Material substitution [tC/ha, a]	Total [tC/ha, a]
Volume strategy	0.8	0.4	1.5	2.3	4.9
Value strategy	0.1	0.4	1.3	2.1	4.0
Carbon storage strategy	1.6	0.1	0.7	1.0	3.4

The 40-year study period (Table 3) shows that in the volume strategy scenario, timber harvest levels in the early stages impact the standing timber volume and therefore reduces the forest carbon stocks. In this case, continually lowering the average age of the remaining stands significantly increases the average yield resulting in a greater amount of available standing timber than in the other two scenarios. Given the greater amount of available wood, this scenario shows a significantly greater C- or CO_2_-effect from wood usage than in the two alternative scenarios. In the carbon storage strategy scenario, forests are managed later in the study period. This is reflected in an increase in forest carbon stocks, a constant C-storage in timber products (HWP carbon stock) and a reduced substitution effect. The C- or CO_2_-effects of wood usage, are, however, much lower, so that in the scenario comparison, the carbon storage strategy scenario has the lowest C- or CO_2_-effect. The volume and value strategy scenarios have similar C- or CO_2_-effects, because the changes in forest carbon stocks balance the effects of wood usage.

The 90 year study period (Table 4) shows significant differences between the scenarios. Wood usage at a later date is no longer able to compensate for the C- or CO_2_-effects of early harvesting. Therefore, the average, annual effect is highest for the volume strategy scenario and lowest for the carbon storage strategy scenario.

The differences between the combined scenarios are, as expected, less significant. The 40-year study period (2011–2050, Table 5) shows an annual climate protection effect similar to the volume and carbon storage strategy scenarios due to the increase in the forest carbon stock and relatively lower levels of wood usage. Over the longer period (2011–2100, Table 6), the average annual climate protection effect of all the combined scenarios is somewhat lower. This is mainly due to the forest’s declining capacity to function as a carbon sink; the increase in deadwood storage up to 2100 cannot compensate for the sharp decline in belowground and aboveground biomass storage.

**Table 5:** Average annual C-effect of forest management and wood usage for the combined scenarios (2011–2050)

	Forest carbon stock [tC/ha, a]	HWP carbon stock [tC/ha, a]	Fuel substitution [tC/ha, a]	Material substitution [tC/ha, a]	Total [tC/ha, a]
Wood use strategy	1.4	0.5	1.1	1.7	4.7
Status quo strategy	2.1	0.4	0.9	1.4	4.8
Conservation strategy	2.7	0.2	0.8	1.1	4.8

**Table 6:** Average annual C-effect of forest management and wood usage for the combined scenarios (2011–2100)

	Forest carbon stock [tC/ha, a]	HWP carbon stock [tC/ha, a]	Fuel substitution [tC/ha, a]	Material substitution [tC/ha, a]	Total [tC/ha, a]
Wood use strategy	0.9	0.3	1.2	1.9	4.4
Status quo strategy	1.0	0.3	1.1	1.7	4.2
Conservation strategy	1.4	0.2	0.9	1.4	3.9

The C- or CO_2_-effects of wood usage are only lower than the C- or CO_2_-effects of the forest storage function in the carbon storage-oriented scenarios (carbon storage strategy and conservation strategy) for the 2011–2050 study period. In all other scenarios and study periods, the C- or CO_2_-effects from wood usage outperform the forest storage function. Table 7 shows for both study periods (until 2050 and 2100) and all scenarios the relative contribution of the forest carbon and the harvested wood products pools as well as the fuel and material substitution.

**Table 7:** Average annual C-effect of forest management and wood usage (2011–2100)

Scenario	Time period	Total effect absolute [tC/ha, a]		Partial effect relative [%]		
			Forest carbon stock	HWP carbon stock	Fuel substitution	Material substitution
Volume strategy	2050	4.7	– 7	20	33	54
	2100	4.9	16	8	30	47
Value strategy	2050	4.8	47	6	19	28
	2100	4.0	3	10	33	54
Carbon storage strategy	2050	4.4	76	0	9	16
	2100	3.4	47	3	21	29
Wood use strategy	2050	4.7	30	11	23	36
	2100	4.4	21	7	28	44
Status quo strategy	2050	4.8	44	8	19	29
	2100	4.2	24	7	27	41
Conservation strategy	2050	4.8	56	4	17	23
	2100	3.9	36	5	23	36

## Discussion

The study shows a cyclic development of carbon storage over time: periods of lower levels of carbon storage follow periods with higher levels. This pattern is the result of the age-class distribution and species diversity found in the individual stands at the baseline year. For the selected study area in Germany, this is due mainly to the period of (re)-afforestation following the Second World War. Forest management scenarios vary between their average long-term carbon stock levels, average harvest levels and the time periods between the phases of higher and lower storage levels. Therefore, the possibilities for assessing the C- or CO_2_-effect of forests based exclusively on changes in their carbon stocks are limited.

Observations based exclusively on forest carbon stocks show that the storage-oriented scenarios (carbon storage strategy, conservation strategy) have a stronger C- or CO_2_-effect than the other scenarios (ecosystem approach). The statement, however, reverses itself if the entire system of carbon stocks in the forest and in HWP, as well as emission reductions through wood usage is considered (sector approach). The climate positive effects of wood usage can already been seen in a 40-year study period, but are even more significant over a longer study period. The scenarios results vary depending on whether the forest management practices are assessed according to the ecosystem approach or the sector approach. The sector approach in contrast to the ecosystem approach takes into account the climate benefits of wood usage which inherently lead to a decline in the forest carbon stock. Since harvesting rejuvenates timber stands and thereby in the long run promotes high growth levels, the sequestration capacity increases, but not to the same overall storage level of an unmanaged forest. The scenario analysis shows that in a comparison between on the one hand an increase in the forest carbon stock and on the other the net effect of the forest’s sink capacity combined with wood usage, managed forests clearly have greater impact. This impact is even more pronounced over longer periods of time and depending on the age structure of stands in the study area.

In the longer study period lasting to 2100, all scenarios show that the C- or CO_2_-effects of wood usage are greater than the C- or CO_2_-effects through the forest’s carbon sink function. This result is in line with the findings by Heuer [25], who calculated that in Germany 84 % of the positive C- or CO_2_-effects are related to wood usage and not to an increase in forest carbon stocks.

Especially the storage-oriented scenarios lead to older stands. In older stands there is a risk of damaging events such as storms or insect calamities that can lead to the reduction of forest carbon stocks and thus the release of C or CO_2_. Studies in Canada and the Bavarian Forest National Park show similar findings among different tree species in their natural habitat, and especially after being put under protection [26, 27]. These risks interrupt the continuous development of the forest carbon stock, but were not accounted for in the study, because no reliable information is available regarding the probability and potential extent of damage.

Uncertainties also exist with regard to C-flows from litter and dead organic material (deadwood) in the soil carbon stock. According to Luyssaert et al. [24] an old stand can still function as a carbon sink even after achieving a balance between biomass accumulation and biomass decomposition, because the C-flow into the soil is ongoing and results in a steady increase in the soil’s C-pool. Luyssaert et al. [19] used a model-based approach without in-situ measurements. Nave et al. [28] used a meta-analysis of 432 data sets on the soil-C response ratio to show that a clear-cutting leads, on average, to an 8 % reduction in soil carbon. Because soil carbon measurements have a high spatial and temporal variability, it is difficult to determine the effects of forest management on soil carbon within a site [29, 30]. The National Inventory Report on the German Greenhouse-Gas Inventory (NIR) [31] clearly shows that a loss in carbon from the forest floor as a result of timber harvesting cannot, at least in Germany, be scientifically documented. The findings of the Second National Forest Soil Inventory (BZE 2) demonstrate that in Germany the soil carbon stocks have stayed the same or even risen [32, 33]. Therefore, in accordance with the IPCC good practice guidance [34], the study at hand also assumes that the soil carbon stock remains constant.

The study area was located in the German state of North Rhine-Westphalia. The scenarios were developed using defined, partially simplifying assumptions. They do not claim to forecast the future of the forest, but serve to identify possible development paths and opportunities. They provide a framework that can serve as a basis for future decision-making parameters to facilitate a strategic approach to climate protection.

## Conclusions

A full assessment of the CO_2_ effects of the forest management and wood-products industries requires an assessment of the forest, i.e., its carbon storage and sink functions, supplemented with an assessment of the effects of wood usage. CO_2_-emission reductions through both fuel and material substitution need to be considered. It is useful to consider this aspect within the framework of the post-Kyoto process.

This study examines the forest management and wood-products industries in the German state of North Rhine-Westphalia. The approach presented, however, is exemplary, and thus transferable to other regions. It can be applied on a small (local) scale as well as over a broader area (e.g., nationally). Thus, the integrated assessment of CO_2_-effects of the forest management and wood utilization based on the developed scenario analysis provides decision-making parameters for a strategic climate protection approach to forest management and wood usage at the regional and national levels.

The short-term evaluation of subsystems can be misleading, rendering long-term evaluations (until 2100, or even longer) more effective. A long-term horizon is also consistent with the inherently long-term perspective of forest management decisions and measures.

## Methods

The model was developed based on the forest management and wood usage data from the German state of North Rhine-Westphalia (NRW; Forest 915,800 ha) [35]. The analysis uses an assessment approach for material flow and carbon flow of the forestry and wood products chain according to [36]. The so-called scenario analysis was used as a methodical approach. The aim was not to forecast the forest’s future as accurately as possible, but to show potential opportunities and future alternative developments [37]. For this purpose, the study defines different assumptions regarding alternative action measures and impacts and formulates these into different scenarios. Using the current status as baseline, these scenarios influence the possible development paths and enable an analysis of the future effects on the forest management and wood-products industries in NRW.

The scenarios were defined based on the following assumptions:

- ▪ Integrated assessment of the system forest development – timber harvest – wood usage.
- ▪ Medium-term (to 2050) and long-term study period (to 2100), to show the forest developments for various scenarios and study periods.
- ▪ The amount of harvested wood is determined by forest management scenarios and not by market demand.
- ▪ Evaluation of emission reductions through material and fuel substitution based on substitution factors, which represent scenarios for wood usage (based on usage scenarios of demand in the German state of North Rhine-Westphalia); assessment of C-sinks in wood products.

### Modeling the CO_2_ impacts of the forest

The forest growth model is based on data from the German National Forest Inventories BWI 1 (1986–1989) and BWI II (2001–2002) [38], timber harvest statistics for the State Forestry Administration of North Rhine-Westphalia as well as yield charts for the main tree species [39–42]. Growth and thinning/harvest rates are based on the yield tables. The growth was corrected for the observed growth taking into account the harvested wood from 1987 to 2002. The carbon balances were derived in line with the IPCC Good Practice Guidance for Land Use, Land Use Change and Forestry [2, 34]. In accordance with the IPCC guidelines (IPCC-GPG), changes were recorded in the five carbon pools (aboveground biomass, belowground biomass, deadwood, litter, soil carbon). In accordance with the IPCC GPG, the model treats carbon stocks in the soil as constant over time (default method). This also applies for the carbon stocks in litter.

The scenario analysis defined three management alternatives (the so-called "basic scenarios"), which, over the long term, all comply with the principle of quantitative sustainability (see Table 8):

**Table 8:** Overview for the definitions for the three basic scenarios

Strategy	Minimum age for usage [years after ATG_max_]		Portion of deadwood / harvest loss [%]	Target level d_1.3_ [cm]		Target stocking level
	hardwoods	softwoods		hardwoods	softwoods	
Volume strategy	0	0	10	-	-	1,0
Value strategy	40	20	20	50	40	1,0
Carbon storage strategy	50	50	40	60	60	1,0

- ▪ volume strategy: strategy with the highest wood production, the rotation period is set to the year of maximum average total growth (ATG_max_)
- ▪ value strategy: strategy focused on long-term timber value appreciation, the rotation period was prolonged and minimum diameter limits, so-called target diameter, were defined. A greater share of harvested wood is left in the forest
- ▪ carbon storage strategy: development of large forest carbon stocks through limited timber harvesting, further prolongation of rotation period, share of residues and target diameter for harvesting increased

The basic scenarios were modeled for the entire forested area in North Rhine-Westphalia and for the duration of the study period. The basic scenarios represent extremes in possible forest management strategies. Under real-world conditions, however, forests are not typically managed to meet one strategic objective, but to fulfill the different interests and objectives of a variety of stakeholders (e.g., forest owners). Therefore, based on the basic scenarios, the study defines three additional combined scenarios – wood use strategy, status quo strategy and conservation strategy – each representing a combination of potential strategic objectives from the basic scenarios applied at varied weights (Table 9). The wood use strategy scenario focuses on intensive wood usage. In the status quo strategy scenario a large forest carbon stock is developed alongside timber harvesting. The conservation strategy scenario reflects extensive increase of forest stocks with the main usage postponed until the trees reach maturity.

**Table 9:** Weighting of the basic scenarios in the combination scenarios

Basic scenarios	Combined scenarios		
	Wood use strategy	Status quo strategy	Conservation strategy
Volume strategy	50.00 %	31.67 %	20.00 %
Value strategy	25.00 %	31.67 %	20.00 %
Carbon stock strategy	20.00 %	31.67 %	50.00 %
Unlogged	5.00 %	5.00 %	10.00 %

While the basic scenarios assume that the entire study area is under management, the combination scenarios also include areas not being used; e.g., unlogged (decommissioned) areas. These areas experience a high level of stand and carbon stock development. Given the absence of reliable data on the growth of unused, former commercial forest, the growth data from yield tables were extrapolated respecting maximum values established by Petritan et al. [43].

The scenarios are based on simple assumptions, for example:

- ▪ The proportions of area per tree species remain constant. Changes in species mix, e.g., from Norway spruce to Douglas fir, or an increased portion of hardwoods were not included in the modeling.
- ▪ The impact of future climate and/or extreme weather events on forest growth and risks (e.g., storms, drought stress) were not included due to the absence of reliable forecasts for such events.
- ▪ A possible increase in growth due to increased CO_2_ concentration in the air was not taken into account which is the conservative approach when forest mitigation potential is assessed.
- ▪ Assuming certain decomposition rates a rough estimate is made for the carbon stock levels of the dead biomass. Given the lack of reliable data for North Rhine-Westphalia, data for Central European and boreal forests (incl. [44–50]) were used to determine an annual decomposition rate of 2.7 % for aboveground biomass and of 4.0 % for belowground biomass.

### Modeling wood usage

The carbon sink capacity of the forest and the annual timber harvest amounts were calculated for each management strategy scenario. Harvested timber amounts were assigned to tree species or wood categories by means of a utilization code. The wood utilization code was regionalized by developing an idealized material-flow model starting with the timber harvest data (based on harvest statistics from 2002–2010) all the way to the final product. The development of the material-flow model was based on national studies on wood usage, e.g., [51], and was adapted to regional conditions based on customer lists complied by the State Forestry Administration, expert opinions and interviews with representatives of the respective industries and associations (such as sawmill industry, pulp and paper industry). In accordance with [11, 52], the end products were classified into product groups with long life spans (sawn timber products, such as construction timber), medium-term life spans (panel material, such as laminate flooring) and short life spans (e.g., paper, packaging materials) as well as fuel wood. For the sake of simplification, it was assumed that wood products exported abroad are used the same way as if they were produced domestically.

Changes in the harvested wood products’ carbon stock are calculated by determining the net input of the carbon in the wood products to the overall HWP carbon pool according to [11, 52].

The **emission reduction** through material and fuel substitution is taken into account at the time the substitution occurs. The calculation of the emission reduction of wood used as fuel is recorded as a credit at the time when the wood is (physically) burned. For wood residues from wood product manufacturing this occurs immediately, for old wood this happens at the time of the End of Life of the respective wood product (recycling is considered).

The **emission reduction through material substitution** is defined using the approach presented by Sathre and O'Connor [22], whereby the difference in CO_2_ emissions (expressed as C) of competing products is set in ratio to their carbon content. Sathre and O'Connor [22] describe this approach for the direct comparison of wood with non-wood products with the same functional units. The literature they evaluated give substitution factors (“displacement factor”) of clearly over 5.0 tC/tC. Certain non-wood products have significantly higher CO_2_ emissions. Sathre and O'Connor's approach, however, only applies to the comparison of two specific products, therefore the mean value of 2.1 tC/tC calculated by them is not always applicable or suitable, it is rather arbitrary based on the studies they selected in their analysis. For a specific case, where a wood market as a whole (as in this case North Rhine-Westphalia) is being analyzed, it is necessary to compare the overall product mix in its whole structure (product types and quantities) as well as the mix of competing products.

In this case the substitution factor SF_Ma_ for material substitution was determined as follows (see also [53]):

1. Initially, 16 key product areas were defined for wood usage and the respective alternative products. Comparisons were made for leading products system, for example parquet, laminate flooring were compared to tiles, PVC or carpet floor (cf. [54]). The product areas cover over 90 % of the wood-usage spectrum. For the 16 product areas, single substitution factors were determined (Table 10; LCA-Basis data of leading products [21, 22]).
2. The 16 product areas were classified taking into account the quantity distribution of wood usage from the material flow analysis by Mantau and Bilitewski [51] for Germany and proportionately attributed to the four product groups construction, furniture, packaging and others which then made it possible to establish a substitution factor (volume-weighted) for each of the four product groups.
3. Based on those four product groups, specific substitution factors and the respective quantitative distribution between those four product groups, a single substitution factor comprising all four product groups was then established/determined to be SF_Ma_ = 1,50 tC/tC. This factor is valid for Germany.

**Table 10:** Analytical approach for determination of substitution factors SF_Ma_ (substitution of material)

Comparison of material systems	SF_Ma_ [tC/tC]
1. Roundwood (poles, fences, buildings, also treated) vs. steel, concrete, aluminum	2.40
2. Softwood lumber, sawn, wet, for packaging concrete shuttering vs. plastics (foils, 3-D elements)	1.80
3. Softwood lumber, planned and dried for building Purposes vs. concrete, steel, bricks	1.40
4. Softwood based glued timber products (glue-lam, CLT) vs. steel, concrete, bricks	1.30
5. Plywood, also overlaid vs. aluminum profiles, glass-fiber-plastic	1.62
6. Wood based panels like particleboard, MDF, OSB (for walls, ceilings, roofs) vs. gypsum board, plaster, concrete, brick type walls	1.10
7. DIY products like lumber, panels, profile boards vs. mineral based products, plastic based panels, aluminum sheets	1.35
8. Wooden flooring (one layer, multi layers), laminate flooring vs. ceramic tiles, plastic flooring, wall to wall carpet	1,35
9. Doors (interior, exterior) – only framing/construction vs. steel, aluminum, PVC	1.62
10. Wooden window frames vs. PVC, aluminum	1.62
11. Wooden furniture (solid wood) vs. glass, plastic, metal	1.62
12. Wooden furniture (panel based) vs. glass, plastics, metal	1.46
13. Wooden kitchen furniture vs. glass, plastics, metal	1.62
14. Other wooden furniture (example: upholstery) vs. glass, plastics, metal	1.62
15. Wood based packaging vs. plastic, metal	1.35
16. Wooden transportation products vs. plastic, metal	1.62

The substitution factor for material substitution of SF_Ma_ = 1,50 tC/tC reflects wood usage in Germany; it was not possible to determine a region-specific substitution factor for NRW due to a lack of precise data on material flow and wood usage and a high degree of variability of those data due to the small market size. It can be assumed, though, that the structure of wood usage in North Rhine-Westphalia does not significantly differ from the structure of wood usage nationwide, based on the comparison of several wood-market parameters between NRW and Germany [55]. The substitution factor of SF_Ma_ = 1,50 tC/tC is, therefore, used as an overall average substitution factor for this study; it was applied for the whole study period until 2100.

In contrary to the typical wood products from mechanical processing a material substitution factor for paper products is not considered because adequate comparative LCA or EPD are not available.

**The emission reduction through fuel substitution** (substitution factor SF_Fuel_) can be calculated based on the difference between the emissions from a defined energy mix of fossil fuels and the emissions from wood, based on its C-content.

The CO_2_-emissions from burning fossil fuels are based on the specific amount of energy (primary or final energy); the CO_2_-emissions from burning wood for energy, on the other hand, only take into account the CO_2_-emissions of fossil fuel used in the earlier parts of the value chain (e.g., use of fossil fuel during forest management, timber harvesting, or transport; see [56]). Besides these steps in the value chain, which use fossil fuel and which overall account for less than 10 % [57]), using wood for energy is thus considered to be CO_2_-neutral. This viewpoint is justified since burning wood only emits as much CO_2_ as has been sequestered from the atmosphere during the life cycle of the trees. Thus, the life cycle of the trees (CO_2_-sink) and burning of the wood (CO_2_-source) offset each other. The fact that the wood removed from the forests through harvest is assessed as CO_2_ emission (excluding the wood which transfers to the harvested wood products pool), makes this approach consistent with the international guidelines of the Kyoto-Protocol [3].

The study uses a substitution factor of SF_Fuel_ = 0.67 tC/tC for fuel substitution. Rüter [58] derives this substitution factor via comparison of the ecological balance of wood with that of light fuel oil as fossil fuel. Taverna et al. [21] determine a substitution factor for fuel substitution for the country of Switzerland of 600 kg CO_2_/m^3^, which corresponds to roughly 0.65 tC/tC. Calculating the substitution factor based on an energy mix of various forms of fossil fuel which would have been burned instead of wood, also confirms a substitution factor of SF_Fuel_ = 0.67 tC/tC [57]. This methodology of referring to fossil fuels is justified as long as fossil fuels are being used, as in North Rhine-Westphalia, which cause CO_2_ emissions which are less or equal to the substitution effect through the emission reduction through the use of wood (cf. [59]). It is expected that this is the case until 2100; therefore the substitution factor is applied for the entire study period.

For the usage of old and used wood it is assumed that only 80 % of the wood is being used for material or fuel use. The remaining 20 % is left unused, as a result for example of decomposition or of being used in open fires without utilization of its thermal energy. It is further assumed that 20 % of the waste wood volume that is being reused, i.e., the amount of wood available at the “end of life stage” is used only once for the production of particleboard, i.e., products with a medium-term life spans.

For pulpwood 85 % energy recovery with a substitution factor of SF_Fuel_ = 0.67 tC/tC is assumed at the end of life of the paper products.
